# Climate and the spread of COVID-19

**DOI:** 10.1038/s41598-021-87692-z

**Published:** 2021-04-27

**Authors:** Simiao Chen, Klaus Prettner, Michael Kuhn, Pascal Geldsetzer, Chen Wang, Till Bärnighausen, David E. Bloom

**Affiliations:** 1 Heidelberg Institute of Global Health, Heidelberg University Medical School, Heidelberg University, Heidelberg, Germany; 2 Chinese Academy of Medical Sciences and Peking Union Medical College, Beijing, China; 3 Department of Economics, Vienna University of Economics and Business, Vienna, Austria; 4 Wittgenstein Centre (IIASA, VID/ÖAW, WU), Vienna Institute of Demography, Vienna, Austria; 5 Economic Frontiers Program, International Institute for Applied Systems Analysis (IIASA) , Laxenburg, Austria; 6 Division of Primary Care and Population Health, Department of Medicine, Stanford University, Stanford, CA USA; 7 National Clinical Research Center for Respiratory Diseases, Beijing, China; 8 Department of Pulmonary and Critical Care Medicine, Center of Respiratory Medicine, China–Japan Friendship Hospital, Beijing, China; 9 Chinese Academy of Engineering, Beijing, China; 10 Africa Health Research Institute (AHRI), Somkhele, KwaZulu-Natal South Africa; 11 Department of Global Health and Population, Harvard T.H. Chan School of Public Health, Boston, MA USA

**Keywords:** Infectious diseases, Environmental social sciences

## Abstract

Visual inspection of world maps shows that coronavirus disease 2019 (COVID-19) is less prevalent in countries closer to the equator, where heat and humidity tend to be higher. Scientists disagree how to interpret this observation because the relationship between COVID-19 and climatic conditions may be confounded by many factors. We regress the logarithm of confirmed COVID-19 cases per million inhabitants in a country against the country’s distance from the equator, controlling for key confounding factors: air travel, vehicle concentration, urbanization, COVID-19 testing intensity, cell phone usage, income, old-age dependency ratio, and health expenditure. A one-degree increase in absolute latitude is associated with a 4.3% increase in cases per million inhabitants as of January 9, 2021 (p value < 0.001). Our results imply that a country, which is located 1000 km closer to the equator, could expect 33% fewer cases per million inhabitants. Since the change in Earth’s angle towards the sun between equinox and solstice is about 23.5°, one could expect a difference in cases per million inhabitants of 64% between two hypothetical countries whose climates differ to a similar extent as two adjacent seasons. According to our results, countries are expected to see a decline in new COVID-19 cases during summer and a resurgence during winter. However, our results do not imply that the disease will vanish during summer or will not affect countries close to the equator. Rather, the higher temperatures and more intense UV radiation in summer are likely to support public health measures to contain SARS-CoV-2.

Given the rapid spread of severe acute respiratory syndrome coronavirus 2 (SARS-CoV-2) in winter 2020/2021 in the Northern Hemisphere, many inhabitants and policymakers in the corresponding countries hope for relieve when the weather gets warmer and more sunlight reaches the Earth’s surface in spring and summer. Indeed, many viral acute respiratory tract infections, such as influenza A and B, rhinovirus, respiratory syncytial virus, adenovirus, metapneumovirus, and coronavirus, are climate dependent and share such seasonal patterns^1^. Some viruses may have better stability in low-temperature, low-humidity, and low-UV radiation environments^2,3^. In addition, people tend to gather more in indoor places in winter, which can facilitate the spread of diseases; and vitamin D levels in humans tend to decline in winter, which may weaken the immune response. Thus, an association between climate conditions and the spread of SARS-CoV-2 seems plausible.

However, in the context of coronavirus disease 2019 (COVID-19), the disease caused by SARS-CoV-2, there is still scant evidence in support of this hypothesis^4^. On March 9, 2020, the World Health Organization (WHO) stated that “from the evidence so far, the COVID-19 virus can be transmitted in all areas, including areas with hot and humid weather”^5^. On April 7, 2020, the U.S. National Academies of Sciences, Engineering, and Medicine concluded that “although experimental studies show a relationship between higher temperatures and humidity levels, and reduced survival of SARS-CoV-2 in the laboratory, there are many other factors besides environmental temperature, humidity, and survival of the virus outside of the host that influence and determine transmission rates among humans in the ‘real world’… with natural history studies, the conditions are relevant and reflect the real-world, but there is typically little control of environmental conditions and there are many confounding factors”^4^.

Between May and November 2020, the European Respiratory Society published several articles discussing the hypothesis that temperature and the spread of COVID-19 are inversely related. Using data from 224 cities in China, one article published in May found no such association^6^. In August 2020, another analysis using data from China implied a non-linear relationship to the extent that temperature and COVID-19 are not associated below 7 ℃ but that a weak negative association exists above that threshold^7^. Yet another study published in November found a significant negative association between temperature and the spread of COVID-19 using global data^8^. While, in general, the evidence is mixed and the debate is still ongoing, laboratory studies found that SARS-CoV-2 is highly susceptible to heat and UV-radiation^9–14^.

To add evidence from a different perspective, we use global data to examine the relationship between climatic conditions and the spread of COVID-19 controlling for several important confounding factors. To this end, we regress the prevalence of COVID-19 (logarithmically transformed) at the country level against the latitude of a country. Latitude captures every climate, because different latitudes on Earth receive different amounts of sunlight. The farther from the equator a country is located, the lower is the angle of the sun’s rays that reach it, the less UV radiation it receives, and the lower is its temperature. Furthermore, latitude also affects humidity, because water evaporation is temperature dependent^15^.

To control for key confounders at the country-level, our analysis includes (1) data on air travel^16^ (to capture a possible way of transmission of SARS-CoV-2 across countries but also the remoteness of a place,  which might increase the need for air travel); (2) vehicle concentration^17^ and urbanization^16^ (to capture differences in the transmission potential of SARS-CoV-2 within a country^18^); (3) COVID-19 testing intensity^19,20^ (to control for the vigor of a country’s COVID-19 response and for COVID-19 detection bias in cross-country comparisons^21,22^); (4) cell phone usage^16^ (to control for the speed at which information on behavior change for COVID-19 prevention travels within a country^18,23^); and (5) health expenditure (to capture differences in countries' commitment to population health); old-age dependency ratio (to capture cross-country differences in age structure and family compositions, which can affect the spread of SARS-CoV-2), and income^16^ (to control for differences in economic development and in the availability of general resources to contain the spread of SARS-CoV-2^24–26^).

## Results

**Figure 1:**
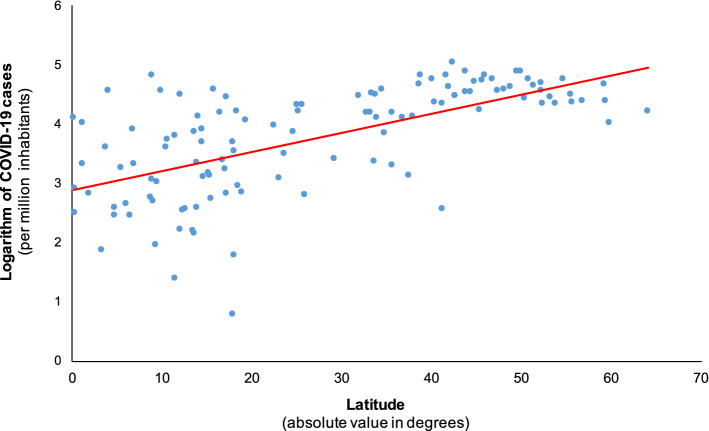
Scatterplot of the natural logarithm of COVID-19 cases per million inhabitants against absolute latitude in degrees for the full sample of countries (R^2^ = 0.40).

Figure 1 and Table 1 show our results. In general, the farther a country is located from the equator, the more cases the country has relative to the number of inhabitants. This relationship is visible in the scatterplot in Fig. 1 and in the coefficient estimates of latitude (which represent semi-elasticities, i.e., percentage changes in the number of COVID-19 cases per million for one-degree changes in latitude), in the different regression specifications shown in Table 1. In the ordinary least squares (OLS) regression, in which we control for all potential confounding factors, an increase in the distance from the equator by one degree of latitude is associated with an increase of the prevalence of COVID-19 by about 4.3% (Table 1, Model 4). This result is highly significant and implies that a country that is located 1000 km closer to the equator could expect 33% fewer cases per million inhabitants, other things equal (given that a degree of latitude translates on average into a distance of 111 km). Since the change in Earth’s angle towards the sun between equinox and solstice is about 23.5°, one could expect a difference in cases per million inhabitants of 64% between two hypothetical countries whose climates differ to a similar extent as two adjacent seasons.

**Table 1:** Results from Ordinary Least Squares regressions of the logarithm of COVID-19 cases per million inhabitants in a country on the country’s latitude and control variables.

	Cases per million inhabitants			
	**(1)** Coefficient 95% CI p value	**(2)** Coefficient 95% CI p value	**(3)** Coefficient 95% CI p value	**(4)** Coefficient 95% CI p value
Latitude	0.071 (0.056–0.086) < 0.001	0.052 (0.032–0.072) < 0.001	0.049 (0.030–0.069) < 0.001	0.043 (0.019–0.067) < 0.001
Air travel		− 0.001 (− 0.060 to 0.058) 0.966	− 0.010 (− 0.073 to 0.0512) 0.738	− 0.046 (− 0.094 to 0.003) 0.065
Vehicle concentration		− 0.237 (− 1.576 to 1.103) 0.726	− 0.841 (− 2.279 to 0.597) 0.248	− 3.288 (− 5.472 to − 1.104) 0.004
Urbanization		0.032 (0.018–0.047) < 0.001	0.029 (0.014–0.044) < 0.001	0.009 (− 0.008 to 0.026) 0.277
Testing intensity			0.006 (− 0.002 to 0.014)	0.002 (− 0.004 to 0.009)
Cell phone usage			0.125 0.009 (0.000–0.017) 0.045	0.472 0.002 (− 0.007 to 0.011) 0.710
Income				1.145 (0.641–1.648) < 0.001
Old-age dependency ratio				− 0.010 (− 0.065 to 0.050) 0.741
Health expenditure				0.167 (0.065–0.269) 0.002
Constant	6.838 (6.242–7.433)	5.464 (4.642–6.285)	4.866 (3.903–5.829)	− 3.803 (− 7.394 to − 0.213)
P value	< 0.001	< 0.001	< 0.001	0.038
*R*^2^	0.428	0.533	0.561	0.643
*Adj. R*^2^	0.423	0.516	0.537	0.614
*N*	117	117	117	117

Column 1 contains the bivariate specification of the regression of the natual logarithm of COVID-19 cases per million inhabitants on latitude. The other columns are nested models with control variables. Models (1) through (4) are alternative specifications and the results are based on countries in which more than 100 cases were reported as of January 9, 2021. Latitude is the absolute latitude of a country in degrees; air travel refers to the number of air passengers per capita in a country; vehicle concentration is the number of registered vehicles per capita; urbanization is the percentage of the population living in cities; testing intensity is the number of tests per hundred inhabitants; cell phone usage refers to the number of cell phones per capita; income refers to the purchasing power-adjusted per-capita gross domestic product (GDP) in a country; old-age dependency ratio is the ratio of the population above the age of 65 to the working-age population; health expenditure refers to the share of GDP spent on health. Robust standard errors are used to account for heteroscedasticity. Missing values were estimated with multiple (15) imputations. CI: confidence interval.

## Discussion

Our results are consistent with the hypothesis that heat and sunlight reduce the spread of SARS-CoV-2 and the prevalence of COVID-19, which was also suggested by most of the previous studies examining the same hypothesis with different data and approaches^8–10,27,28^. However, our results do not imply that the disease will vanish during summer. Rather, the higher temperatures and more intense UV radiation in summer are likely to support public health measures to contain SARS-CoV-2^29,30^. WHO’s warning that the virus spreads in all climates must still be taken seriously. At the time of revising this manuscript in January 2021, many countries in the Northern Hemisphere are experiencing a surge in COVID-19 cases, which could be explained by an easier spread of COVID-19 in winter.

Our analysis has several limitations. First, while our results are consistent with the hypothesis that higher temperatures and more intense UV radiation reduce SARS-CoV-2 transmission, the precise mechanisms for such an effect remain unclear and may indeed comprise not only biological but also behavioral factors. For example, people might gather less in crowded indoor places if temperatures are higher – a behavior reducing transmission. Thus, future research should aim at uncovering how the transmission of SARS-CoV-2 is affected by changes in (1) climatic factors such as heat and humidity, (2) geographic factors such as altitude and sunlight intensity, (3) factors related to human behavior such as social interactions and pollution due to local economic activity at a more disaggregated level, and (4) the different potential of the human immune system to cope with diseases in summer as opposed to winter. Second, even though we included all countries worldwide for which data for this analysis were available, our final data set included only 117 out of the world’s countries, mainly for reasons of data availability and for some countries not yet having surpassed the 100 COVID-19 case threshold. Third, while we strived to control for differential testing intensity using a recently compiled and frequently updated data set^19,20^, the data on testing intensity could suffer from reporting biases and incomplete coverage of testing approaches. To the extent that testing intensity is a function of a country’s income, our analysis controlling for income (Table 1, Model 4) should reduce such a bias. The fact that column (4) in Table 1 contains a parameter estimate of latitude that is only slightly lower than the one in column (3) and still highly significant is reassuring in this regard. Furthermore, factors such as health infrastructure, socioeconomic background, and the availability of adequate health supplies may also affect the spread of COVID-19. However, these differences can be at least partially captured by controlling – as we have done – for vehicle concentration, urbanization, cell phone usage, income, the old-age dependency ratio, health expenditure, and testing intensity. Fourth, we cannot, as of yet, assess whether mutated versions of SARS-CoV-2, such as the ones that emerged in South Africa or in the UK in fall 2020, will display similar seasonal patterns of infection. Finally, the distance to the equator has the same climatic effects going north and south only when we are either around equinox or when one full year in the pandemic has passed (such that the seasonal variations average out globally because both hemispheres have passed through all four seasons during the pandemic). Thus, the date of our data set (which we updated during the final revision of this manuscript in January 2021) is comparatively well-suited for our analysis, because at this point in time the COVID-19 pandemic had been spreading for approximately 1 year^31,32^. Moreover, the effect sizes we estimate stayed rather stable over time. In earlier analyses of the data in March and April 2020^33^, which is close to equinox, we also found a significant positive association between latitude and the number of cases. Since then, the semi-elasticity estimates increased slightly, which could be due to better data quality and larger numbers of observations in our updated data sets.

In sum, we show that an increase in absolute latitude by 1° is associated with a 4.3% increase in COVID-19 cases per million inhabitants. Increasing temperatures and longer sunlight exposure during summer may boost the impact of public health policies and actions to control the spread of SARS-CoV-2. Conversely, the threat of epidemic resurgence may increase during winter. However, our results do not indicate that the disease will vanish in summer, nor that countries located close to the equator will contain the disease without effective public health measures.

## Methods

We estimated both the bivariate specification of the regression of the logarithm of COVID-19 cases per million inhabitants on latitude as well as nested models with control variables. We excluded countries in which less than 100 COVID-19 cases were reported as of January 9, 2021, to use only data from countries where the pandemic was spreading (a few cases could be merely imported). Our main exposure variable is the absolute latitude of a country in degrees. The control variables included: (1) air travel, measured by the number of air passengers per capita in a country; (2) vehicle concentration, measured by the number of registered vehicles per capita; (3) urbanization, measured by the percentage of the population living in cities; (4) testing intensity, measured by the number of tests per hundred inhabitants; (5) cell phone usage, measured by the number of cell phones per capita; (6) income, measured by purchasing power-adjusted per-capita gross domestic product in a country; (7) old-age dependency ratio, which is the ratio of the population above the age of 65 to the working-age population; (8) health expenditure, which is the share of per-capita GDP spent on health. We used 2018 data for air travel, vehicle concentration, income, urbanization, cell phone usage, old-age dependency ratio, and health expenditure, because more recent data were not available in the World Development Indicators, our data source for these variables^16^. Testing intensity was based on testing data gathered for each country^19,20^. We used robust standard errors to account for heteroscedasticity. We used Stata 16 for our multivariable regression analyses. We estimated missing country covariate data in multiple (15) imputations, using the *mibeta* Stata command^34^.

## Supplementary information

Supplementary Information.

## Data Availability

All data are available in the main text or the supplementary materials.
