# Supplementary material for: Climate and the spread of COVID-19
Source: Sci Rep. 2021 Apr 27;11:9042. doi: 10.1038/s41598-021-87692-z (PMC8079387; doi:10.1038/s41598-021-87692-z)
Supplement: Supplementary file 1 — Supplementary Information. [file 41598_2021_87692_MOESM1_ESM.zip › Paper title and author.docx]

Climate and the spread of COVID-19

**Authors:** Simiao Chen^1,2,†^, Klaus Prettner^3,4†^, Michael Kuhn^4^, Pascal Geldsetzer^1,5^, Chen Wang^2,6,7,8,*^, Till Bärnighausen^1,2,9,10, ‡^, David E. Bloom^10,#,*,‡^

**Affiliations:**

^1^ Heidelberg Institute of Global Health, Heidelberg University Medical School, Heidelberg University, Heidelberg, Germany.

^2^ Chinese Academy of Medical Sciences & Peking Union Medical College, Beijing, China.

^3^ Vienna University of Economics and Business, Department of Economics, Vienna, Austria.

^4^ Wittgenstein Centre (IIASA, VID/ÖAW, WU), Vienna Institute of Demography, Vienna, Austria.

^5^ Division of Primary Care and Population Health, Department of Medicine, Stanford University, Stanford, CA, USA.

^6^ National Clinical Research Center for Respiratory Diseases, Beijing, China.

^7^ Department of Pulmonary and Critical Care Medicine, Center of Respiratory Medicine, China–Japan Friendship Hospital, Beijing, China.

^8^ Chinese Academy of Engineering, Beijing, China.

^9^ Africa Health Research Institute (AHRI), Somkhele, KwaZulu-Natal, South Africa

^10^ Department of Global Health and Population, Harvard T.H. Chan School of Public Health, Boston, MA, USA.

† Co-first authors: SC and KP contributed equally to this study

‡ Co-senior authors: TB and DB

* Co-corresponding authors:

DB: [dbloom@hsph.harvard.edu](mailto:dbloom@hsph.harvard.edu)

CW: [wangchen@pumc.edu.cn](mailto:wangchen@pumc.edu.cn)
